# A Novel Modified Omega-K Algorithm for Synthetic Aperture Imaging Lidar through the Atmosphere

**DOI:** 10.3390/s8053056

**Published:** 2008-05-06

**Authors:** Liang Guo, Mendao Xing, Yu Tang, Jing Dan

**Affiliations:** 1 The National Key Laboratory of Radar Signal Processing, Xidian University, Xi'an, P.R. China E-mails: lguo@mail.xidian.edu.cn; xmd@xidian.edu.cn; 2 The National Key Laboratory of Radar Signal Processing, Xidian University, Xi'an, P.R. China E-mails: tangyu0905@yahoo.com.cn; 411jingdan@163.com

**Keywords:** Synthetic aperture, lidar, FMCW, Omega-K algorithm, phase screens, coherence length of atmosphere

## Abstract

The spatial resolution of a conventional imaging lidar system is constrained by the diffraction limit of the telescope's aperture. The combination of the lidar and synthetic aperture (SA) processing techniques may overcome the diffraction limit and pave the way for a higher resolution air borne or space borne remote sensor. Regarding the lidar transmitting frequency modulation continuous-wave (FMCW) signal, the motion during the transmission of a sweep and the reception of the corresponding echo were expected to be one of the major problems. The given modified Omega-K algorithm takes the continuous motion into account, which can compensate for the Doppler shift induced by the continuous motion efficiently and azimuth ambiguity for the low pulse recurrence frequency limited by the tunable laser. And then, simulation of Phase Screen (PS) distorted by atmospheric turbulence following the von Karman spectrum by using Fourier Transform is implemented in order to simulate turbulence. Finally, the computer simulation shows the validity of the modified algorithm and if in the turbulence the synthetic aperture length does not exceed the similar coherence length of the atmosphere for SAIL, we can ignore the effect of the turbulence.

## 1. Introduction

A conventional optical imager is limited in spatial resolution by the diffraction limit of the telescope aperture [1]. In practice there are many factors to that limit attempts to improve the resolution by increasing the telescope aperture. Synthetic aperture (SA) techniques that are well known in microwave imaging can increase the resolution beyond the diffraction limit of the receiving aperture. And the spatial terminal resolution of synthetic aperture radar in azimuth is limited by the wavelength of the carrier. Consequently research on the application of SA techniques to the optical domain, enabling fine-resolution, two-dimensional, active imaging at a long range with small diameter optics is of great value.

A number of fundamental but innovative synthetic-aperture experiments in the optical domain were performed [2-4]. Some prior research relates to SA processing in optical wavelengths for rotating objects like inverse synthetic aperture radar (ISAR) in microwave [2]. Some experiments in the principle of synthetic aperture radar (SAR) were successfully demonstrated in laboratory [3]. If we want to bring the experiments to the outside for long-range application, e.g., air-borne or space-borne synthetic aperture imaging lidar (SAIL), many effects should be taken into account [5].

Fortunately, we can learn experience from SAR, a mature field that was developed to construct microwave images of high resolution by use of antennas of reasonable size. The spotlight model SAR allows the generation of images with a high geometry resolution. If we make SAIL work in spotlight model, amazing resolution can be obtained. Since the SAIL system uses continuous wave (CW) form while pulses are used in the SAR system [6], we can not directly make use of conventional SAR techniques like the model that Karr has described in his analysis [7]. And as a result of the continuous wave, we can not provide a high pulse recurrence frequency (PRF), which will lead to azimuth ambiguity [8] in the spotlight model of the SAIL system.

In this paper, the FM-CW SAIL signal processing will be addressed and the response to a point target will be developed first. Then, a modified Omega-K algorithm will be derived for the CW signal in the spotlight model. Finally, computer simulation will show the validity of the modified algorithm. Considering the effect of the atmosphere that certainly has potential to do harm to SAIL resolution, the wave distortions caused by atmospheric turbulence will be simulated by means of phase screens, and analysis in provided.

## 2. FMCW SAIL signal and definition of imaging geometry

This section derives an analytical development of the dechirped signal [here by “dechirped” we mean heterodyning the return chirped signal with a similarly chirped local oscillator (LO)], which is the FMCW SAIL signal in the two-dimensional time domain without using the stop-and-go approximation that is assumed in a conventional pulsed SAR system.

**Figure 1.**
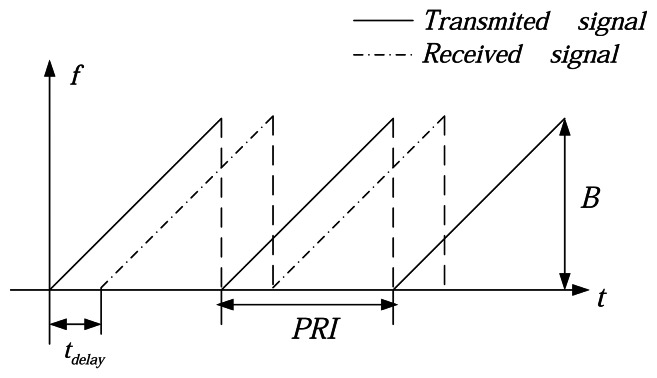
FMCW signal in frequency-time domain.

Figure 1 shows the frequency versus time characteristics of the transmitted signal (solid line) and the received signal (dashed line).The received signal is a delayed version of the transmitted one. The lidar continuously transmits linear FM chirps with duration *T_p_* equal to the pulse repetition interval *PRI* and the reciprocal of *PRF*. The transmitted signal is expressed as

(1) $$s_{T}=A\cdot \text{rect}\left[\frac{\hat{t}}{T_{p}}\right]\exp\left(j2\pi (f_{c}+\frac{1}{2}\gamma {\hat{t}}^{2})\right)$$

where −*T_p_* /2 ≤ *t̂* < *T_p_* /2, the chirp rate *γ*= *B*/ *PRI*, where *B* is the transmitted bandwidth and *f_c_* is the center frequency. The envelope of the transmitted pulse and the antenna diagram are included in the parameter A with a constant phase but a varying amplitude.

For typical pulsed SAR systems the pulse length *T_p_* is sufficiently short that the radar is assumed stationary during the transmission and the reception of the signal. This is the so-called “stop-and-go” approximation to the case when the platform, such as an aircraft, is flying. It appears as if it stopped, sent a pulse, received it and then moved to the next position. Conventional SAR algorithms make use of this assumption. If the duration of the pulse is increased, the approximation can not be considered valid any more. In the case of FM-CW SAIL, this approximation may no longer be valid because relatively long sweeps are transmitted.

**Figure 2.**
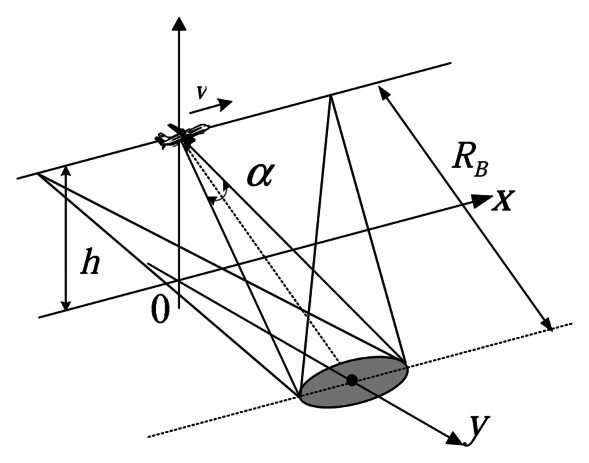
SAIL system geometry.

Figure 2 shows the basic geometry of a spotlight mode of a SAIL system. The platform with a transmitter–receiver module moves across the synthetic aperture, namely the azimuth direction, with speed *v* perpendicular to the line of sight (LOS), namely the range direction. The beamwidth is *a*, the height of the platform is *h*, the range from the target center to the sampling aperture pupil antenna phase center (APC) at the closest approach is *R_B_*, and *R_t_* is the sampling-aperture-pupil-dependent distance to the point target:

(2) $$R_{t}=R_{t}\left(\hat{t},t_{m}\right)=\sqrt{R_{B}^{2}+{\left(v\hat{t}+vt_{m}\right)}^{2}}$$

where, *vt̂* indicates that the stop-and-go approximation is not valid any more, since *R_t_* depends only on *t_m_* in a conventional SAR system. Here *t_m_* = *nT_p_*, namely the time of the recording during the synthetic aperture time *T_a_*, i.e. $t_{m}\in \left[-\frac{T_{a}}{2},\frac{T_{a}}{2}\right]$. And considering the effect of the atmosphere, we can obtain the value of the phase error through the turbulence in the same way as in [9, 10].

(3) $$\begin{array}{ll} \phi \left(x,y\right) & =F\{\Phi (f_{x},f_{y})\}\\ & =F\left\{0.00058\;r_{0}^{-5/3}{\left(f_{x}^{2}+f_{y}^{2}+f_{0}^{2}\right)}^{-11/6}\right\} \end{array}$$

where, *F* is the Fourier transform, Φ(*f_x_*, *f_y_*) is the power spectral density of the refractive index fluctuations of the atmosphere (using the von Karman model) and *r̃*_0_ is the similar coherence length of atmosphere defined by Karr [4]. The variables *f_x_* and *f_y_* are the spatial frequencies. *f*_0_ = 2*π* / *L*_0_, and *L*_0_ is called the outer scale. Due to the fact that the phase error is a nonparametric error, it is hard for us to give the exact expression. In the simulation, we transform the phase error *φ*(*x*,*y*) to the variable Δ*r* by $\Delta r=\frac{\phi \left(x,y\right)}{2\pi }\lambda$. Therefore $R_{t}^{\prime }=R_{t}+\Delta r$, and $R_{t}^{\prime }$ is the real distance form the pupil to the point, which is like the way of dealing with the motion of the platform [6]. Here, we ignore the phase error (if we choose a right synthetic aperture length) in order to simplify the derivation. Then we replace $R_{t}^{\prime }$ by *R_t_* in the subsequent section, which dose not affect the analysis.

So the received signal can be written as

(4) $$s_{R}=A\cdot \text{rect}\left(\frac{\hat{t}-\tau }{T_{p}}\right)\exp\left(j2\pi \left(f_{c}(t-\tau )+\frac{1}{2}\gamma {\left(\hat{t}-\tau \right)}^{2}\right)\right)$$

which is a replica of the transmitted signal delayed by *τ*, where $\tau =\frac{2R_{t}}{c}$, *c* is the speed of light, and 2 stands for the round-trip. In the same way, the reference signal can be written as:

(5) $$s_{\text{ref}}=A\cdot \text{rect}\left(\frac{\hat{t}-\tau_{\text{ref}}}{T_{p}}\right)\cdot \exp\left(j2\pi \left(f_{c}(t-\tau_{\text{ref}})+\frac{1}{2}\gamma {\left(\hat{t}-\tau_{\text{ref}}\right)}^{2}\right)\right)$$

where $\tau_{\text{ref}}=\frac{2R_{\text{ref}}}{c}$, and *R_ref_* could be zero or an arbitrary but fixed value. In this paper, we choose *R_s_* as the slant range distance of the scene center at boresight position as *R_ref_*. In this heterodyne detection system, the received and the referenced signal (which can be the transmitted signal or its delayed one) are mixed. In the receiver, the received signal is demodulated with the reference signal. The demodulation results in the beat signal

(6) $$\begin{array}{ll} s_{\text{beat}} & =s_{R}\cdot s_{\text{ref}}^{*}\\ & =A\cdot \text{rect}\left(\frac{\hat{t}-2R_{t}/c}{T_{p}}\right)\cdot \exp\left(-j\frac{4\pi R_{t}}{\lambda }\right)\cdot \exp\left(-j\frac{4\pi \gamma }{c}\left(R_{t}-R_{s}\right)(\hat{t}-\frac{2R_{\text{ref}}}{c})\right)\cdot \exp\left(j\frac{4\pi \gamma }{c^{2}}{\left(R_{t}-R_{s}\right)}^{2}\right) \end{array}$$

In the equation, the first exponential term is the Doppler modulation in azimuth and the second term represents the range signal, which is a sinusoidal signal with a constant frequency corresponding to the azimuth dependent distance to the point target *R_t_*, and the last exponential term represents the residual video phase (RVP) term, and can be ignored for its long sweeps, i.e., a small chirp rate.

Starting from the square root expression for range Equation (2), it is useful to make the approximation:

(7) $$R_{t}=R_{t}\left(\hat{t},t_{m}\right)\approx R\left(t_{m}\right)+\frac{v^{2}t_{m}}{R\left(t_{m}\right)}\hat{t}$$

where $R\left(t_{m}\right)=\sqrt{R_{B}^{2}+{\left(vt_{m}\right)}^{2}}$. This approximation is valid for a CW model, since the terms higher than the second order in the series expansion (7) are very small. Then the Doppler shift effect, induced by the continuous motion within the sweep, can be expressed as:

(8) $$f_{d}=-\frac{2}{\lambda }\cdot \frac{dR_{t}}{d\hat{t}}=-\frac{2}{\lambda }\cdot \frac{v^{2}t_{m}}{R\left(t_{m}\right)}=\frac{2}{\lambda }\cdot v\cdot \cos\theta =f_{a}$$

where *λ* is the carrier wavelength, *θ* is the Doppler cone angle between the antenna velocity vector and the radar line of sight to the target center, and *f_a_* is the Doppler frequency in azimuth right equal to the Doppler shift *f_d_* which must be compensated, otherwise it will affect image location and geometric fidelity, and result in distortion.

In a SAIL system, the effects induced by the continuous motion are very small in $\exp\left(-j\frac{4\pi }{c}\gamma \left(R_{t}-R_{s}\right)\left(\hat{t}-\frac{2R_{s}}{c}\right)\right)$, so they can be replaced by $\exp\left(-j\frac{4\pi }{c}\gamma \left(R\left(t_{m}\right)-R_{s}\right)\left(\hat{t}-\frac{2R_{s}}{c}\right)\right)$ in Equation (6). So ignoring RVP through range deskew, the signal becomes:

(9) $$s\left(\hat{t},t_{m}\right)=A\cdot \text{rect}\left(\frac{\hat{t}-2R_{s}/C+2v^{2}t_{m}/\gamma \lambda R}{T_{p}}\right)\cdot \exp\left(-j\frac{4\pi }{\lambda }\frac{v^{2}t_{m}}{R\left(t_{m}\right)}\hat{t}\right)\cdot \exp\left(-j\frac{4\pi \gamma }{c}\left(R\left(t_{m}\right)-R_{s}\right)(\frac{f_{c}}{\gamma }+\hat{t}-\frac{2R_{s}}{c})\right)$$

The term $\frac{2v^{2}t_{m}}{\gamma \lambda R}$ of the envelope is small compared to the delay of the signal and can be ignored.

The fist exponential term is the Doppler shift induced by continuous motion. Here, we make use of the following several simplifying assumptions, all of which can be eliminated by suitable methods,

- The laser is frequency chirped for range resolution.
- The laser is modeled as a scalar wave field (no optical polarization).
- The image effects of noise, laser speckle [11].

## 3. Modified Omega-K algorithm

### 3.1 Azimuth preprocessing

First of all, we take the azimuth ambiguity mentioned above into account. As we know, the ambiguity is induced by a smaller PRF than the azimuth bandwidth in the spotlight case. A way to overcome this limitation of PRF is based on the spectral analysis technique [8]. The aim of this preprocessing is to eliminate the azimuth ambiguity rather than focus the image. So we just need to pay attention to the second exponential term $\left(-j\frac{4\pi f_{c}}{c}R\left(t_{m}\right)\right)$ in Equation (9). The received signal can be represented in a simplified form by [12] using Equation (7):

(10) $$s_{a}=\text{rect}\left(\frac{t_{m}-t_{m,\text{center}}}{T_{a}}\right)\cdot \exp\left(-j\frac{2v^{2}}{\lambda R_{B}}t_{m}^{2}\right)=\text{rect}\left(\frac{t_{m}-t_{m,\text{center}}}{T_{a}}\right)\cdot \exp\left(-jk_{a}t_{m}^{2}\right)$$

where *t*_*m,center*_ is the center of *T_a_*, and $k_{a}=\frac{2v^{2}}{\lambda R_{B}}$, namely the azimuth chirp rate [6].

The key step of azimuth preprocessing is a convolution operation between the signal *s_a_* in (10) and the following quadratic phase signal:

(11) $$s_{a,\text{ref}}=\exp\left(-jk_{a,\text{ref}}t_{m}^{2}\right)$$

This convolution operation can be represented by:

(12) $$s_{\text{co}n\text{v}}=s_{a}⊗s_{a,\text{ref}}=\exp\left(jk_{a,\text{ref}}t_{m}^{2}\right)\int s_{a}\cdot \exp\left(jk_{a,\text{ref}}\tau^{2}\right)\cdot \exp\left(-j2\pi k_{a,\text{ref}}\tau t_{m}\right)d\tau$$

And leads to a bulk azimuth raw data compression that reduces the azimuth signal bandwidth.

Let us now consider the discrete domain implementation of the azimuth preprocessing step, which is particularly due to the sampled characteristics of the raw signal, and rewrite the Equation (12) as follows:

(13) $$\begin{array}{l} s_{\text{co}n\text{v}}\left(n\Delta t^{\prime \prime },R_{B}\right)=\sum_{i=-Q/2}^{Q/2-1}s_{a}\left(i\Delta t^{\prime },R_{B}\right)s_{a,\text{ref}}\left(n\Delta t^{\prime \prime }-i\Delta t^{\prime },R_{\text{ref}}\right)\\ =\exp\left(jk_{a,\text{ref}}{\left(n\Delta t^{\prime \prime }\right)}^{2}\right)\sum_{i=-Q/2}^{Q/2-1}s_{a}\left(i\Delta t^{\prime },R_{B}\right)\exp\left(jk_{a,\text{ref}}{\left(i\Delta t^{\prime }\right)}^{2}\right)\exp\left(-j2\pi k_{a,\text{ref}}n\Delta t^{\prime \prime }i\Delta t^{\prime }\right)\\ n=-B/2,\ldots ,B/2-1 \end{array}$$

where Δ*t*′ = 1/ *PRF*, the original pulse repetition interval, *Q* is the raw signal azimuth pixel number, Δ*t*″ is the pulse repetition interval of the output signal, and *B* is the output signal azimuth pixel number. If we assume:

(14) $$\begin{array}{ccc} \frac{1}{k_{a}\Delta t^{\prime }}=P\Delta t^{\prime \prime }, & \text{and} & P\in N \end{array}$$

**Figure 3.**
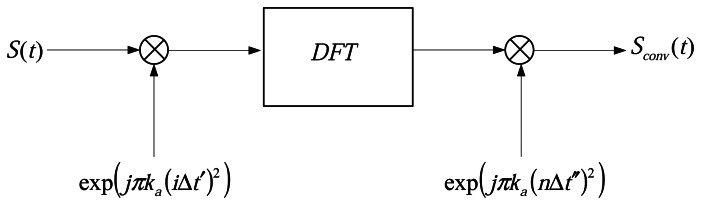

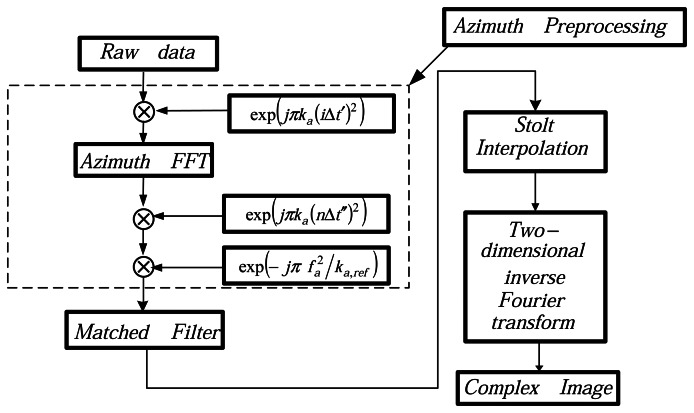
Sketch of azimuth preprocessing. The data processing using the modified Omega-K algorithm.

We finally rewrite eq. (13) as follows:

(15) $$\begin{array}{l} s_{\text{co}n\text{v}}\left(n\Delta t^{\prime \prime },R_{B}\right)=\exp\left(jk_{a,\text{ref}}{\left(n\Delta t^{\prime \prime }\right)}^{2}\right)\text{DFT}\left[s_{a}\left(i\Delta t^{\prime },R_{B}\right)\exp\left(jk_{a,\text{ref}}{\left(i\Delta t^{\prime }\right)}^{2}\right)\right]\\ n=-P/2,\ldots ,P/2-1 \end{array}$$

where the symbol *DFT* [·] represents the discrete Fourier transform operator. In a word, this operation can be carried out by the simple multiplication and a discrete Fourier transform, and the block diagram showing the azimuth preprocessing implemented in Equation (10) is presented in Figure 3.

Up to now, the azimuth ambiguity has been eliminated, but in the operation a convolution has been done. Now we must countervail the convolution by multiplying the following equation:

(16) $$H\left(f_{a}\right)=\exp\left(j\pi \frac{f_{a}^{2}}{k_{a,\text{ref}}}\right)$$

where *f_a_* is the Doppler frequency in azimuth, namely the Doppler frequency, varying within the following range: $-\frac{\text{PRF}}{2}+f_{dc}\leq f_{a}\leq +\frac{\text{PRF}}{2}+f_{dc}$, and here PRF is the pulse repetition frequency, i.e., the reciprocal of PRI. In the sidelooking condition, *f_dc_* is zero; otherwise it is a fixed value which depends on the Doppler cone angle at the closest approach. *k_a,ref_* is the same as *k_a,ref_* in (11) and similar with *k_a_* in (10) corresponding to *R_ref_* (namely *R_s_*) not *R_B_*. At this point, the azimuth preprocessing is done completely. It is worth noting that the minimal PRF must be greater than the bandwidth corresponding to the width of the illuminated field by the laser beam.

### 3.2 Modified Omega-K algorithm

The first operation of the algorithm is a Fourier transform in the along-track dimension [13, 14]:

(17) $$s\left(\hat{t},f_{a};R_{B}\right)=\text{Arect}\left(\frac{\hat{t}-2R_{s}/C}{T_{p}}\right)\cdot \exp\left(j\frac{4\pi }{\lambda }R_{s}\left(1+\frac{\lambda \gamma }{c}\left(\hat{t}-\frac{2R_{s}}{c}\right)\right)\right)\cdot \exp\left(j2\pi f_{a}\hat{t}\right)\cdot \exp\left(-j\frac{4\pi R_{B}}{\lambda }\sqrt{{\left(1+\frac{\lambda \gamma }{c}\left(\hat{t}-\frac{2R_{s}}{c}\right)\right)}^{2}-\frac{f_{a}^{2}\lambda^{2}}{4v^{2}}}\right)$$

Different from [10, 11], the second exponential term in the expression (17) is the Doppler shift induced by the long sweeps of a continuous wave.

The second step in the Omega-K algorithm is application of a two-dimensional phase compensation to the azimuth-transformed signal using the following matched filter:

(18) $$H=\exp\left(-j2\pi f_{a}\hat{t}\right)\cdot \exp\left(-j\frac{4\pi }{\lambda }R_{s}\left(1+\frac{\lambda \gamma }{c}\left(\hat{t}-\frac{2R_{s}}{c}\right)\right)\right)\cdot \exp\left(j\frac{4\pi R_{s}}{\lambda }\sqrt{{\left(1+\frac{\lambda \gamma }{c}\left(\hat{t}-\frac{2R_{s}}{c}\right)\right)}^{2}-\frac{f_{a}^{2}\lambda^{2}}{4v^{2}}}\right)$$

This operation perfectly corrects the range curvature of all scatterers at the same range as the scene center and removes the Doppler shift due to the long sweeps of the continuous wave successively.

Then, the received signal is obtained as:

(19) $$s\left(\hat{t},f_{a};R_{B}\right)=A\cdot \exp\left(-j\frac{4\pi \left(R_{B}-R_{s}\right)}{\lambda }\sqrt{{\left(1+\frac{\lambda \gamma }{c}\left(\hat{t}-\frac{2R_{s}}{c}\right)\right)}^{2}-\frac{f_{a}^{2}\lambda^{2}}{4v^{2}}}\right)$$

The third step performed by the Omega-K algorithm is known as Stolt transformation [15-17]. We can make the following substitutions for convenience [16].

(20) $$K_{R}=\frac{4\pi \gamma }{c}\left(\frac{f_{c}}{\gamma }+\left(\hat{t}-\frac{2R_{s}}{c}\right)\right)$$

and

(21) $$K_{X}=\frac{2\pi f_{a}}{v}$$

With these definitions (19) becomes:

(22) $$s\left(K_{X},K_{R};R_{B}\right)=\text{Arect}\left[\frac{K_{R}-4\pi f_{c}/c}{4\pi \gamma T_{p}/c}\right]\exp\left(-j\left(R_{B}-R_{s}\right)\sqrt{K_{R}^{2}-K_{X}^{2}}\right)$$

At this point, *s*(*K_X_*, *K_R_*; *R_B_*) is a two-dimensional linear phase grating for all scatterers in the same range bin as the scene center(all the scatterers having the same *R_B_*).A change of variables, known as the Stolt interpolation, converts *s*(*K_X_*, *K_R_*; *R_B_*) into a linear phase grating for scatterers at all ranges. The appropriate change of variables is:

(23) $$\sqrt{K_{R}^{2}-K_{X}^{2}}\to K_{Y}$$

Application of this transformation yields the desired signal:

(24) $$s\left(K_{X},K_{R};R_{B}\right)=\text{Arect}\left[\frac{K_{R}-4\pi f_{c}/c}{4\pi \gamma T_{p}/c}\right]\exp\left(-j\left(R_{B}-R_{s}\right)K_{Y}\right)$$

Finally, a two-dimensional inverse Fourier transform is computed to fully compress the scatterers in range and azimuth. Figure 3 shows the data processing using the modified Omega-K algorithm.

## 3. Experimental Results

SAIL has a very different parametric dependence from real-aperture (active or passive) imaging on the usual optical parameters such as aperture *D* or wavelength *λ*. Table 1 shows the parameters of the simulation.

**Figure 4.**
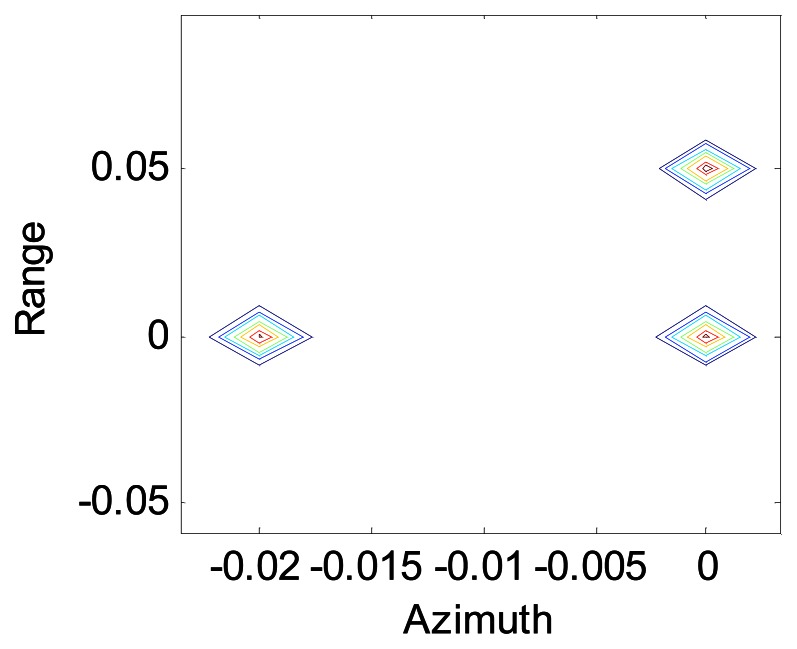
Contour of the points.

Figure 4 shows the image of a scene composed of three point objects, one at (0,0) m, one at (0.02,0) m and one at (0,0.05) m after SA processing using the modified Omega-K algorithm, and we can see that the points are all focused in range and azimuth.

**Figure 5.**
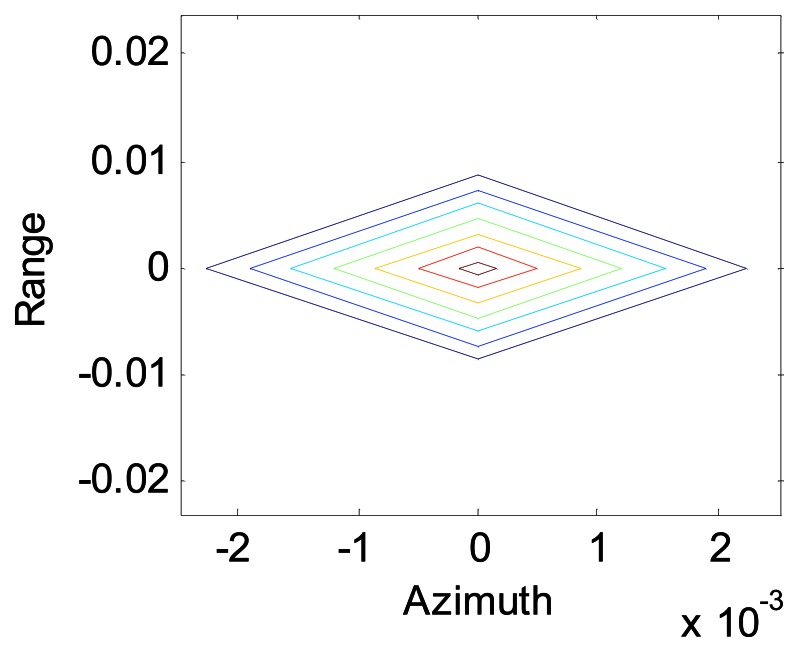
Contour map of one point dimension

**Figure 6.**
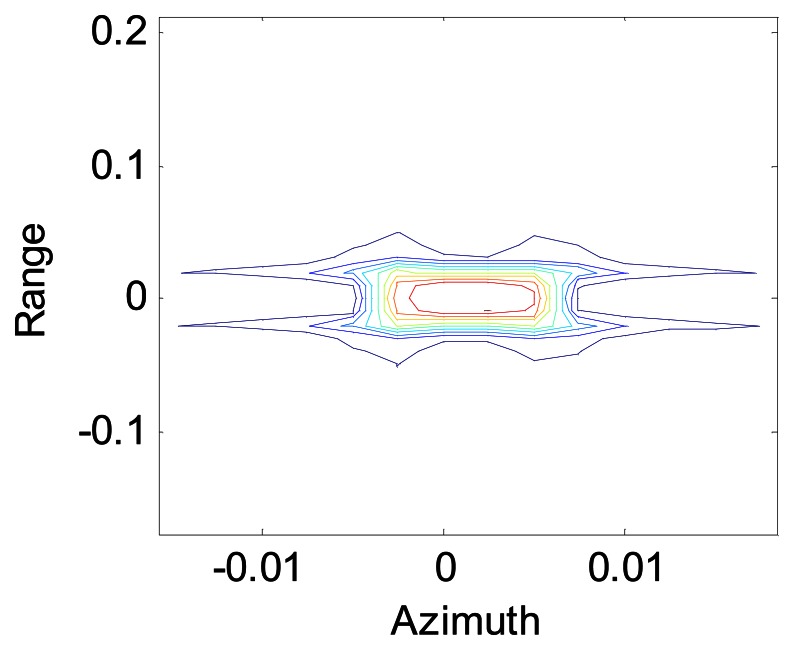
Profile of azimuth dimension.

Figures 5 and 6 show the contour maps of a point after and before the Doppler shift compensation respectively. Figure 5 shows the data processing after using the modified Omega-K algorithm, while Figure 6 represents the data processing after using the conventional Omega-K algorithm. We can see that the data defocus if the Doppler shift induced by the continuous motion of the platform in the sweeps is not compensated.

**Figure 7.**
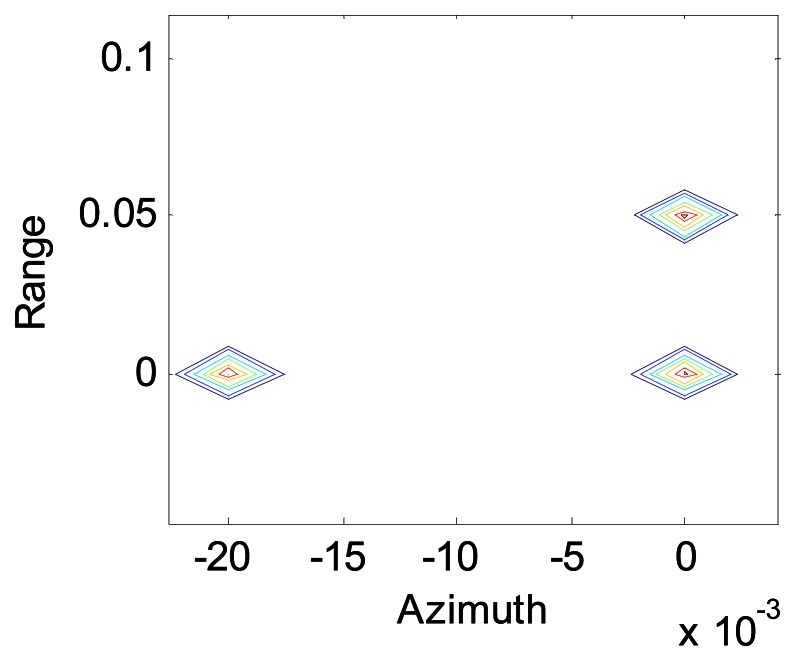
ratio of synthetic aperture length to coherence length of atmosphere is 1/4.

Figure 7 shows the image of a scene composed of three point objects, one at (0,0) m, one at (0.02,0) m and one at (0,0.05) m, after SA processing for a fairly low level of turbulence, and it seems that there was no turbulence like Figure 4. The loss of resolution is negligible when *r̃*_0_ = 3.2*m* for this case, while the length of the synthetic aperture is *L_SA_* = 0.8*m*.

**Figure 8.**
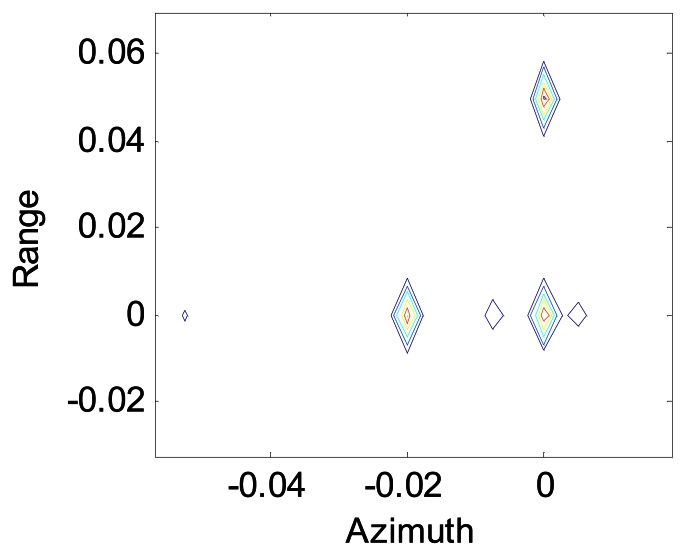
ratio of synthetic aperture length to coherence length of atmosphere is 1/2.

Figure 8 shows the image of the same scene with a little higher turbulence; *r̃*_0_ = 1.6*m* in this case and *L_SA_*/*r̃*_0_ = 1/2. The sidelobes become higher, and they come out pseudo-objects. But we can still recognize the point objects, and the resolution in range is unaffected.

**Figure 9.**
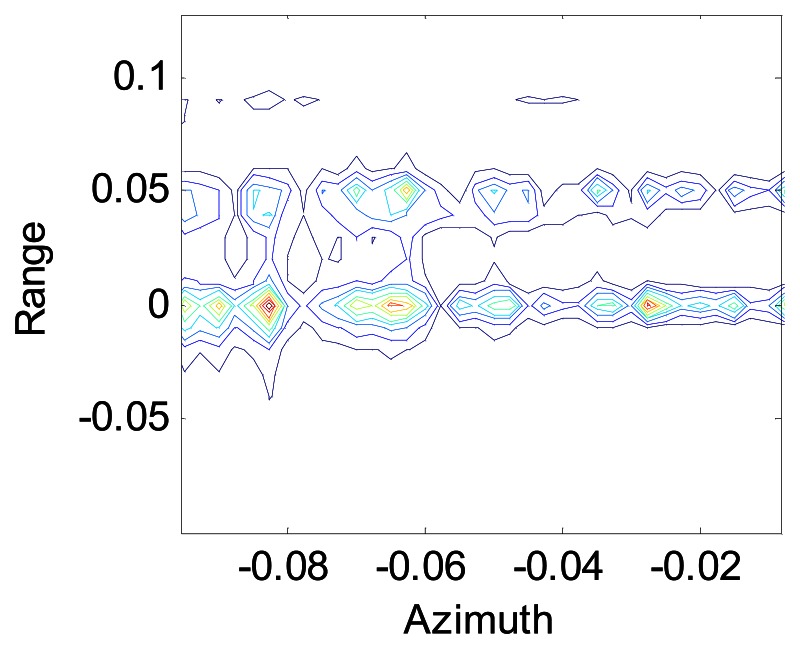
ratio of synthetic aperture length to coherence length of atmosphere is 8/1.

Figure 9 shows the image of the same scene with fairly high turbulence; *r̃*_0_ = 0.1*m* in this case and *L_SA_*/*r̃*_0_ = 8. We can see that image quality is severely degraded, but the resolution in range is also unaffected.

## 5. Conclusions

In summary, we have derived a modified frequency algorithm that is suitable for the SAIL in spotlight model using the heterodyne detection system and some results of a simulation of SAIL through the turbulence have been given. We know that SA processing is feasible at the optical wavelength, and that the given algorithm can process the data successfully. SAIL range resolution is unaffected by turbulence, while SAIL azimuth resolution is determined not only by the synthetic aperture length [6] but also by the coherence length of atmosphere, which is identical with the result given by Karr [7]. Clearly, there are still many challenges that need to be overcome to put this technique into practice, but the reasonable algorithm of the synthetic aperture technique for 2-D imaging in the optical domain is now given. Other models of SAIL will be studied and analyzed in the future, such as the spotlight model with a fixed squint angle, and many effects such as the photon limitation would be taken into consideration in the future work.

## Figures and Tables

**Table 1.** SAIL and phase screen simulation parameters.

Wavelength *λ*	1*μm*
Bandwidth B	15*GH_Z_*
Pulse repetition interval *PRI*	200*μs*
Distance between platform and center of the imaging area *R_s_*	4000*m*
Velocity *v*	50*m*/ *s*
Sampling aperture in azimuth D	2*cm*
Width of the area *W_r_*	10*m*
Sampling frequency *F_s_*	300*MH_Z_*
Grid interval	0.01*mm*
Grid point number	512×512
Outer scale *L*_0_	20*m*
